# Supplementary material for: Interferon alpha-inducible protein 6 regulates NRASQ61K-induced melanomagenesis and growth
Source: eLife. 2016 Sep 8;5:e16432. doi: 10.7554/eLife.16432 (PMC5031487; doi:10.7554/eLife.16432)
Supplement: Supplementary file 1. — (A) List of genes that are significantly upregulated (p<0.05, Fold-change = 2 fold or more) in NRASQ61K expressing MEL-ST cells. (B) Fold change (FC) for significantly altered genes (p-value<0.05) in YUGASP cells expressing IFI6 shRNAs. (C) Biological pathway enrichment analysis report. (D) Primer sequences for RT-qPCR analysis; clone ID and catalog numbers for shRNAs (Open Biosystems); antibodies used; source and concentration of chemical inhibitors used. DOI: http://dx.doi.org/10.7554/eLife.16432.031 [file elife-16432-supp1.docx]

**Supplementary File legends**

**Supplementary File 1**

(**A**) List of genes that are significantly upregulated (p<0.05, Fold-change = 2 fold or more) in NRASQ61K expressing MEL-ST cells. (**B**) Fold change (FC) for significantly altered genes (p-value <0.05) in YUGASP cells expressing IFI6 shRNAs. (**C**) Biological pathway enrichment analysis report. (**D**) Primer sequences for RT-qPCR analysis; clone ID and catalog numbers for shRNAs (Open Biosystems); antibodies used; source and concentration of chemical inhibitors used.

**Supplementary file 1A:** List of genes that are significantly upregulated ( p<0.05, Fold-change = 2 fold or more) in NRASQ61K expressing MEL-ST cells

| **Gene Symbol** | **Gene Name** | **Fold change** | **adj.P.Val** |
| --- | --- | --- | --- |
| IFI27 | interferon, alpha-inducible protein 27 | 71.6524756 | 4.42E-17 |
| IL8 | interleukin 8 | 64.09082051 | 4.81E-11 |
| IL1B | interleukin 1, beta | 49.73598698 | 2.75E-13 |
| IL8 | interleukin 8 | 41.74475592 | 8.93E-09 |
| MX1 | myxovirus (influenza virus) resistance 1, interferon-inducible protein p78 (mouse) | 36.41615974 | 4.42E-17 |
| IFI6 | interferon, alpha-inducible protein 6 | 34.62442864 | 1.36E-15 |
| PTGS2 | prostaglandin-endoperoxide synthase 2 (prostaglandin G/H synthase and cyclooxygenase) | 32.13919526 | 1.36E-15 |
| DCN | decorin | 31.63680731 | 3.15E-12 |
| IL6 | interleukin 6 (interferon, beta 2) | 29.44040612 | 5.11E-10 |
| CXCL1 | chemokine (C-X-C motif) ligand 1 (melanoma growth stimulating activity, alpha) | 26.26708175 | 6.52E-11 |
| OAS2 | 2'-5'-oligoadenylate synthetase 2, 69/71kDa | 17.74699014 | 2.24E-16 |
| KYNU | kynureninase | 13.96962038 | 1.42E-11 |
| CCL2 | chemokine (C-C motif) ligand 2 | 13.96394567 | 1.09E-09 |
| IFITM1 | interferon induced transmembrane protein 1 (9-27) | 13.96154143 | 1.60E-14 |
| C1QTNF1 | C1q and tumor necrosis factor related protein 1 | 13.71086288 | 3.31E-11 |
| ISG15 | ISG15 ubiquitin-like modifier | 13.4369812 | 3.08E-13 |
| IFI6 | interferon, alpha-inducible protein 6 | 13.27004242 | 5.74E-08 |
| IFI44L | interferon-induced protein 44-like | 12.98171747 | 1.82E-12 |
| PTGS2 | prostaglandin-endoperoxide synthase 2 (prostaglandin G/H synthase and cyclooxygenase) | 12.83306051 | 6.95E-10 |
| CCL20 | chemokine (C-C motif) ligand 20 | 10.47326286 | 8.33E-09 |
| CCL5 | chemokine (C-C motif) ligand 5 | 9.891729115 | 5.79E-11 |
| IL24 | interleukin 24 | 9.583320154 | 5.07E-13 |
| PTGES | prostaglandin E synthase | 9.194147949 | 5.51E-10 |
| HLA-B | major histocompatibility complex, class I, B | 9.04219577 | 1.88E-11 |
| IFIT1 | interferon-induced protein with tetratricopeptide repeats 1 | 8.902339179 | 1.42E-11 |
| EPSTI1 | epithelial stromal interaction 1 (breast) | 8.872973251 | 1.76E-12 |
| TNFRSF21 | tumor necrosis factor receptor superfamily, member 21 | 8.848787238 | 1.19E-12 |
| CXCL5 | chemokine (C-X-C motif) ligand 5 | 8.384939112 | 3.29E-08 |
| HLA-F | major histocompatibility complex, class I, F | 7.680770403 | 2.13E-12 |
| MX2 | myxovirus (influenza virus) resistance 2 (mouse) | 7.666325887 | 5.72E-09 |
| COL7A1 | collagen, type VII, alpha 1 | 7.662684816 | 1.24E-08 |
| OASL | 2'-5'-oligoadenylate synthetase-like | 7.620627026 | 4.44E-11 |
| MMP3 | matrix metallopeptidase 3 (stromelysin 1, progelatinase) | 7.443128686 | 4.51E-13 |
| DCN | decorin | 7.316170706 | 1.33E-06 |
| BST2 | bone marrow stromal cell antigen 2 | 7.313149079 | 1.60E-09 |
| TYMP | thymidine phosphorylase | 6.722160789 | 5.72E-09 |
| ISG20 | interferon stimulated exonuclease gene 20kDa | 6.668371651 | 1.78E-14 |
| SOD2 | superoxide dismutase 2, mitochondrial | 6.656417224 | 8.53E-13 |
| HLA-H | major histocompatibility complex, class I, H (pseudogene) | 6.622823668 | 2.13E-12 |
| CXCL5 | chemokine (C-X-C motif) ligand 5 | 6.577342995 | 1.58E-09 |
| DNER | delta/notch-like EGF repeat containing | 6.562463447 | 4.81E-11 |
| TNFAIP6 | tumor necrosis factor, alpha-induced protein 6 | 6.418975416 | 9.58E-11 |
| CXCL2 | chemokine (C-X-C motif) ligand 2 | 6.412271702 | 2.28E-08 |
| IFI44 | interferon-induced protein 44 | 6.307394788 | 3.53E-09 |
| NFKBIZ | nuclear factor of kappa light polypeptide gene enhancer in B-cells inhibitor, zeta | 6.293973138 | 8.17E-12 |
| IL11 | interleukin 11 | 6.246253874 | 3.40E-09 |
| CCL5 | chemokine (C-C motif) ligand 5 | 6.162792226 | 1.20E-09 |
| OAS1 | 2'-5'-oligoadenylate synthetase 1, 40/46kDa | 5.951096526 | 1.44E-09 |
| C1S | complement component 1, s subcomponent | 5.862553451 | 3.48E-10 |
| SOD2 | superoxide dismutase 2, mitochondrial | 5.814046904 | 3.48E-10 |
| PID1 | phosphotyrosine interaction domain containing 1 | 5.808749995 | 1.70E-10 |
| COL10A1 | collagen, type X, alpha 1 | 5.780284408 | 2.45E-07 |
| SLC16A6 | solute carrier family 16, member 6 (monocarboxylic acid transporter 7) | 5.739416182 | 5.08E-07 |
| IFIT2 | interferon-induced protein with tetratricopeptide repeats 2 | 5.52672756 | 6.29E-08 |
| KYNU | kynureninase | 5.502980083 | 6.94E-09 |
| IFIT3 | interferon-induced protein with tetratricopeptide repeats 3 | 5.454457769 | 6.95E-10 |
| EGR1 | early growth response 1 | 5.432546081 | 1.10E-09 |
| CFB | complement factor B | 5.365958117 | 5.49E-08 |
| OAS3 | 2'-5'-oligoadenylate synthetase 3, 100kDa | 5.365067107 | 2.01E-11 |
| NA | NA | 5.355807173 | 4.81E-11 |
| STAT1 | signal transducer and activator of transcription 1, 91kDa | 5.349656213 | 2.31E-12 |
| DPYSL3 | dihydropyrimidinase-like 3 | 5.252050295 | 2.85E-12 |
| NFKBIA | nuclear factor of kappa light polypeptide gene enhancer in B-cells inhibitor, alpha | 5.218263103 | 1.43E-10 |
| XAF1 | XIAP associated factor 1 | 5.087237157 | 3.04E-10 |
| C1R | complement component 1, r subcomponent | 5.062544711 | 2.47E-10 |
| ANPEP | alanyl (membrane) aminopeptidase | 4.847903122 | 1.57E-06 |
| OAS1 | 2'-5'-oligoadenylate synthetase 1, 40/46kDa | 4.821300452 | 1.03E-08 |
| NDRG1 | N-myc downstream regulated 1 | 4.770074394 | 6.72E-11 |
| NA | NA | 4.757615359 | 7.09E-11 |
| HERC5 | hect domain and RLD 5 | 4.680985683 | 4.82E-11 |
| HERC6 | hect domain and RLD 6 | 4.667298287 | 2.90E-09 |
| IFITM3 | interferon induced transmembrane protein 3 | 4.606663974 | 1.48E-09 |
| C1R | complement component 1, r subcomponent | 4.596921838 | 1.20E-09 |
| HCP5 | HLA complex P5 (non-protein coding) | 4.451435916 | 2.31E-12 |
| OAS1 | 2'-5'-oligoadenylate synthetase 1, 40/46kDa | 4.376272068 | 1.06E-09 |
| KRT81 | keratin 81 | 4.346879675 | 8.93E-09 |
| SMOX | spermine oxidase | 4.345734212 | 1.44E-06 |
| LY6E | lymphocyte antigen 6 complex, locus E | 4.326210638 | 1.36E-07 |
| GPAM | glycerol-3-phosphate acyltransferase, mitochondrial | 4.32435071 | 5.21E-09 |
| KIAA1199 | KIAA1199 | 4.225645118 | 6.59E-09 |
| ANGPTL4 | angiopoietin-like 4 | 4.222762849 | 2.03E-08 |
| GPAM | glycerol-3-phosphate acyltransferase, mitochondrial | 4.211792376 | 1.50E-09 |
| TAP1 | transporter 1, ATP-binding cassette, sub-family B (MDR/TAP) | 4.194498431 | 6.30E-09 |
| NA | NA | 4.193484424 | 2.90E-09 |
| STAT1 | signal transducer and activator of transcription 1, 91kDa | 4.142794806 | 2.64E-09 |
| NA | NA | 4.133544683 | 1.42E-11 |
| IRF9 | interferon regulatory factor 9 | 4.129086794 | 3.87E-10 |
| FPR1 | formyl peptide receptor 1 | 4.094858396 | 1.24E-10 |
| TFPI2 | tissue factor pathway inhibitor 2 | 4.06582768 | 2.96E-07 |
| ZC3H12A | zinc finger CCCH-type containing 12A | 4.062414752 | 2.15E-09 |
| HLA-H | major histocompatibility complex, class I, H (pseudogene) | 4.040600577 | 1.43E-10 |
| OAS2 | 2'-5'-oligoadenylate synthetase 2, 69/71kDa | 4.029735125 | 1.08E-10 |
| PAPPA | pregnancy-associated plasma protein A, pappalysin 1 | 3.960954964 | 3.78E-08 |
| SLC25A24 | solute carrier family 25 (mitochondrial carrier; phosphate carrier), member 24 | 3.951550344 | 1.17E-06 |
| SMOX | spermine oxidase | 3.928507885 | 8.30E-09 |
| NTSR1 | neurotensin receptor 1 (high affinity) | 3.892070767 | 3.87E-10 |
| CDKN1A | cyclin-dependent kinase inhibitor 1A (p21, Cip1) | 3.872650784 | 3.59E-10 |
| ITGA2 | integrin, alpha 2 (CD49B, alpha 2 subunit of VLA-2 receptor) | 3.857100926 | 4.81E-11 |
| TMEM132A | transmembrane protein 132A | 3.839712467 | 4.92E-06 |
| GBP2 | guanylate binding protein 2, interferon-inducible | 3.827503444 | 1.27E-11 |
| TNFRSF19 | tumor necrosis factor receptor superfamily, member 19 | 3.74651057 | 6.11E-08 |
| RGS2 | regulator of G-protein signaling 2, 24kDa | 3.675685429 | 1.72E-09 |
| HLA-A | major histocompatibility complex, class I, A | 3.662273708 | 3.65E-11 |
| THBS2 | thrombospondin 2 | 3.661020458 | 2.90E-11 |
| IFIT3 | interferon-induced protein with tetratricopeptide repeats 3 | 3.648074907 | 5.69E-05 |
| SERPINE2 | serpin peptidase inhibitor, clade E (nexin, plasminogen activator inhibitor type 1), member 2 | 3.635189952 | 3.48E-10 |
| FST | follistatin | 3.595721137 | 3.79E-07 |
| PRIC285 | peroxisomal proliferator-activated receptor A interacting complex 285 | 3.593408088 | 8.10E-10 |
| HLA-C | major histocompatibility complex, class I, C | 3.589446873 | 3.96E-11 |
| IFIH1 | interferon induced with helicase C domain 1 | 3.573529381 | 1.55E-07 |
| LGALS3BP | lectin, galactoside-binding, soluble, 3 binding protein | 3.5676426 | 6.99E-07 |
| C15orf48 | chromosome 15 open reading frame 48 | 3.554565928 | 7.43E-12 |
| CXCL6 | chemokine (C-X-C motif) ligand 6 (granulocyte chemotactic protein 2) | 3.486527469 | 6.36E-06 |
| SCG5 | secretogranin V (7B2 protein) | 3.475426809 | 8.26E-11 |
| IFITM2 | interferon induced transmembrane protein 2 (1-8D) | 3.465733323 | 6.30E-09 |
| IRF7 | interferon regulatory factor 7 | 3.459068834 | 5.24E-09 |
| TIMP1 | TIMP metallopeptidase inhibitor 1 | 3.452656732 | 7.38E-10 |
| RUNX3 | runt-related transcription factor 3 | 3.417675229 | 2.42E-08 |
| TNIP1 | TNFAIP3 interacting protein 1 | 3.367932008 | 1.93E-07 |
| CSF2 | colony stimulating factor 2 (granulocyte-macrophage) | 3.361942994 | 3.19E-08 |
| HLA-F | major histocompatibility complex, class I, F | 3.334795748 | 1.42E-11 |
| COL6A1 | collagen, type VI, alpha 1 | 3.333156369 | 1.75E-06 |
| IL24 | interleukin 24 | 3.287769403 | 2.00E-06 |
| HCG4 | HLA complex group 4 (non-protein coding) | 3.266396832 | 8.06E-09 |
| RSAD2 | radical S-adenosyl methionine domain containing 2 | 3.261760026 | 2.14E-09 |
| QPCT | glutaminyl-peptide cyclotransferase | 3.258137627 | 3.29E-07 |
| TRIB1 | tribbles homolog 1 (Drosophila) | 3.257181371 | 2.47E-09 |
| AKR1B1 | aldo-keto reductase family 1, member B1 (aldose reductase) | 3.247097658 | 1.67E-09 |
| NA | NA | 3.221494398 | 3.82E-11 |
| NA | NA | 3.203018581 | 4.31E-08 |
| IER3 | immediate early response 3 | 3.188688635 | 3.13E-09 |
| VCAM1 | vascular cell adhesion molecule 1 | 3.16961704 | 5.49E-09 |
| WISP1 | WNT1 inducible signaling pathway protein 1 | 3.167418723 | 4.88E-09 |
| STC1 | stanniocalcin 1 | 3.162494636 | 7.19E-10 |
| XAF1 | XIAP associated factor 1 | 3.125853499 | 3.21E-08 |
| PFKFB4 | 6-phosphofructo-2-kinase/fructose-2,6-biphosphatase 4 | 3.109034854 | 7.51E-09 |
| PARP12 | poly (ADP-ribose) polymerase family, member 12 | 3.090690252 | 6.22E-07 |
| IGFBP3 | insulin-like growth factor binding protein 3 | 3.077616281 | 4.43E-11 |
| PCDH7 | protocadherin 7 | 3.063622847 | 2.47E-10 |
| PSMB9 | proteasome (prosome, macropain) subunit, beta type, 9 (large multifunctional peptidase 2) | 3.062870555 | 6.57E-09 |
| IFI35 | interferon-induced protein 35 | 3.061099469 | 1.85E-07 |
| ACTG2 | actin, gamma 2, smooth muscle, enteric | 3.02131094 | 9.88E-12 |
| AGRN | agrin | 3.014224498 | 2.07E-05 |
| NBL1 | neuroblastoma, suppression of tumorigenicity 1 | 3.000415906 | 1.89E-05 |
| STAT1 | signal transducer and activator of transcription 1, 91kDa | 2.99381258 | 6.57E-09 |
| NBL1 | neuroblastoma, suppression of tumorigenicity 1 | 2.971368343 | 1.85E-05 |
| FAM46A | family with sequence similarity 46, member A | 2.966734944 | 1.90E-09 |
| APCDD1L | adenomatosis polyposis coli down-regulated 1-like | 2.949021747 | 4.38E-11 |
| FHOD3 | formin homology 2 domain containing 3 | 2.94858374 | 3.39E-11 |
| NR4A2 | nuclear receptor subfamily 4, group A, member 2 | 2.94337856 | 1.11E-07 |
| GAS1 | growth arrest-specific 1 | 2.938381429 | 0.000185977 |
| MDK | midkine (neurite growth-promoting factor 2) | 2.913175333 | 1.25E-06 |
| HTRA1 | HtrA serine peptidase 1 | 2.908653308 | 1.06E-06 |
| SAMD9 | sterile alpha motif domain containing 9 | 2.907240518 | 1.84E-08 |
| GPR68 | G protein-coupled receptor 68 | 2.906035333 | 2.62E-08 |
| ITPKA | inositol-trisphosphate 3-kinase A | 2.901977218 | 2.87E-10 |
| COL6A2 | collagen, type VI, alpha 2 | 2.887838852 | 0.001209977 |
| GPR183 | G protein-coupled receptor 183 | 2.882208642 | 8.22E-08 |
| COL4A1 | collagen, type IV, alpha 1 | 2.866788792 | 2.09E-07 |
| BHLHE40 | basic helix-loop-helix family, member e40 | 2.861285667 | 3.44E-10 |
| ABCC3 | ATP-binding cassette, sub-family C (CFTR/MRP), member 3 | 2.843348601 | 9.89E-08 |
| LAMA5 | laminin, alpha 5 | 2.83730864 | 1.76E-08 |
| HLA-A | major histocompatibility complex, class I, A | 2.817116646 | 0.000270075 |
| LIF | leukemia inhibitory factor (cholinergic differentiation factor) | 2.805846754 | 8.86E-08 |
| PARP9 | poly (ADP-ribose) polymerase family, member 9 | 2.804215686 | 2.64E-07 |
| CEBPB | CCAAT/enhancer binding protein (C/EBP), beta | 2.802003629 | 1.68E-08 |
| CHST15 | carbohydrate (N-acetylgalactosamine 4-sulfate 6-O) sulfotransferase 15 | 2.795312071 | 1.02E-08 |
| NA | NA | 2.78536948 | 4.49E-05 |
| CABLES1 | Cdk5 and Abl enzyme substrate 1 | 2.782205421 | 8.20E-07 |
| CSF3 | colony stimulating factor 3 (granulocyte) | 2.781016917 | 5.22E-06 |
| ABCA1 | ATP-binding cassette, sub-family A (ABC1), member 1 | 2.777857257 | 8.93E-09 |
| GLIS3 | GLIS family zinc finger 3 | 2.776788619 | 6.13E-10 |
| IFIT3 | interferon-induced protein with tetratricopeptide repeats 3 | 2.773060334 | 7.99E-06 |
| PMAIP1 | phorbol-12-myristate-13-acetate-induced protein 1 | 2.73149654 | 2.27E-06 |
| IGFBP3 | insulin-like growth factor binding protein 3 | 2.701616682 | 2.15E-09 |
| SAMD9L | sterile alpha motif domain containing 9-like | 2.683545875 | 9.90E-08 |
| STAT2 | signal transducer and activator of transcription 2, 113kDa | 2.679089994 | 1.38E-07 |
| TMEM158 | transmembrane protein 158 (gene/pseudogene) | 2.672675599 | 2.04E-09 |
| SPOCK1 | sparc/osteonectin, cwcv and kazal-like domains proteoglycan (testican) 1 | 2.662131233 | 1.22E-09 |
| TRIM22 | tripartite motif containing 22 | 2.660560335 | 4.40E-06 |
| RASD1 | RAS, dexamethasone-induced 1 | 2.651390478 | 4.81E-11 |
| HES4 | hairy and enhancer of split 4 (Drosophila) | 2.644226771 | 7.46E-09 |
| UCN2 | urocortin 2 | 2.638145002 | 1.30E-05 |
| TSKU | tsukushi small leucine rich proteoglycan homolog (Xenopus laevis) | 2.632483309 | 1.74E-10 |
| RHOB | ras homolog gene family, member B | 2.630062232 | 2.36E-10 |
| FBLN2 | fibulin 2 | 2.621616277 | 3.29E-08 |
| FBLN2 | fibulin 2 | 2.610765401 | 8.61E-09 |
| DDX60 | DEAD (Asp-Glu-Ala-Asp) box polypeptide 60 | 2.608444672 | 1.06E-05 |
| SLC25A37 | solute carrier family 25, member 37 | 2.599664519 | 9.93E-06 |
| B2M | beta-2-microglobulin | 2.594462676 | 5.31E-06 |
| KLF9 | Kruppel-like factor 9 | 2.579531768 | 5.13E-06 |
| GNG11 | guanine nucleotide binding protein (G protein), gamma 11 | 2.573572614 | 1.15E-05 |
| COL6A2 | collagen, type VI, alpha 2 | 2.571020359 | 0.000522277 |
| OASL | 2'-5'-oligoadenylate synthetase-like | 2.566778286 | 7.64E-08 |
| CDCP1 | CUB domain containing protein 1 | 2.565840474 | 9.11E-06 |
| NA | NA | 2.560886673 | 1.01E-08 |
| S100A4 | S100 calcium binding protein A4 | 2.558649546 | 6.60E-09 |
| NAMPT | nicotinamide phosphoribosyltransferase | 2.557912101 | 6.87E-05 |
| HLA-G | major histocompatibility complex, class I, G | 2.556406246 | 3.58E-09 |
| HAS1 | hyaluronan synthase 1 | 2.517093511 | 6.96E-08 |
| SLC39A8 | solute carrier family 39 (zinc transporter), member 8 | 2.510078898 | 1.05E-05 |
| STMN3 | stathmin-like 3 | 2.493297081 | 2.46E-08 |
| SRPX | sushi-repeat containing protein, X-linked | 2.487690274 | 3.14E-08 |
| LRRN3 | leucine rich repeat neuronal 3 | 2.477583892 | 1.03E-09 |
| EIF2AK2 | eukaryotic translation initiation factor 2-alpha kinase 2 | 2.472581398 | 9.18E-06 |
| IRF7 | interferon regulatory factor 7 | 2.46971623 | 1.91E-06 |
| UBE2L6 | ubiquitin-conjugating enzyme E2L 6 | 2.463279639 | 1.73E-10 |
| C13orf33 | chromosome 13 open reading frame 33 | 2.446585383 | 2.14E-06 |
| CEBPD | CCAAT/enhancer binding protein (C/EBP), delta | 2.445712986 | 8.86E-08 |
| NA | NA | 2.444112536 | 7.38E-08 |
| SH2B3 | SH2B adaptor protein 3 | 2.439701681 | 8.08E-08 |
| EDNRA | endothelin receptor type A | 2.429041735 | 1.80E-09 |
| NA | NA | 2.42409914 | 2.42E-08 |
| FAP | fibroblast activation protein, alpha | 2.422418878 | 1.22E-08 |
| PTPRE | protein tyrosine phosphatase, receptor type, E | 2.418651744 | 7.19E-10 |
| S100A4 | S100 calcium binding protein A4 | 2.412475414 | 6.94E-08 |
| CD82 | CD82 molecule | 2.409138817 | 8.24E-07 |
| DDIT4 | DNA-damage-inducible transcript 4 | 2.407739333 | 2.34E-08 |
| NAMPT | nicotinamide phosphoribosyltransferase | 2.402599764 | 0.017570299 |
| DUSP6 | dual specificity phosphatase 6 | 2.401984152 | 4.96E-08 |
| B2M | beta-2-microglobulin | 2.396574364 | 3.04E-06 |
| PLSCR1 | phospholipid scramblase 1 | 2.392043524 | 0.000796468 |
| TNFAIP3 | tumor necrosis factor, alpha-induced protein 3 | 2.384751382 | 8.95E-09 |
| IGFBP5 | insulin-like growth factor binding protein 5 | 2.380687865 | 1.84E-06 |
| TNFRSF1B | tumor necrosis factor receptor superfamily, member 1B | 2.372219865 | 2.38E-07 |
| SLC15A3 | solute carrier family 15, member 3 | 2.362720514 | 1.84E-08 |
| SLC22A23 | solute carrier family 22, member 23 | 2.359771597 | 2.78E-08 |
| TMEM51 | transmembrane protein 51 | 2.351533036 | 3.21E-08 |
| SH3PXD2A | SH3 and PX domains 2A | 2.344942152 | 2.04E-09 |
| CXCL6 | chemokine (C-X-C motif) ligand 6 (granulocyte chemotactic protein 2) | 2.33801592 | 4.73E-07 |
| RARRES3 | retinoic acid receptor responder (tazarotene induced) 3 | 2.336178172 | 1.51E-05 |
| PMEPA1 | prostate transmembrane protein, androgen induced 1 | 2.334095044 | 7.29E-09 |
| SLC22A4 | solute carrier family 22 (organic cation/ergothioneine transporter), member 4 | 2.321613395 | 8.35E-07 |
| ANKRD29 | ankyrin repeat domain 29 | 2.319969474 | 3.52E-07 |
| CRIP2 | cysteine-rich protein 2 | 2.314620202 | 6.40E-06 |
| RSPO3 | R-spondin 3 | 2.309756984 | 3.65E-08 |
| SH3PXD2B | SH3 and PX domains 2B | 2.30677815 | 4.67E-06 |
| COL5A2 | collagen, type V, alpha 2 | 2.304440248 | 4.16E-09 |
| IRAK2 | interleukin-1 receptor-associated kinase 2 | 2.301179508 | 1.88E-05 |
| NA | NA | 2.288821228 | 1.06E-07 |
| PNKD | paroxysmal nonkinesigenic dyskinesia | 2.275520899 | 9.57E-07 |
| SLC22A4 | solute carrier family 22 (organic cation/ergothioneine transporter), member 4 | 2.263537108 | 2.34E-08 |
| ARID3A | AT rich interactive domain 3A (BRIGHT-like) | 2.261516615 | 5.30E-06 |
| IQCD | IQ motif containing D | 2.259162337 | 2.96E-07 |
| REC8 | REC8 homolog (yeast) | 2.257208823 | 3.90E-06 |
| DUSP4 | dual specificity phosphatase 4 | 2.254529776 | 8.44E-06 |
| UBA7 | ubiquitin-like modifier activating enzyme 7 | 2.254173841 | 8.86E-08 |
| CD68 | CD68 molecule | 2.254017326 | 4.96E-08 |
| PLAU | plasminogen activator, urokinase | 2.251395862 | 7.92E-08 |
| C3 | complement component 3 | 2.25098724 | 1.21E-08 |
| FAM108C1 | family with sequence similarity 108, member C1 | 2.248398028 | 7.52E-08 |
| PRRX1 | paired related homeobox 1 | 2.246871232 | 1.06E-06 |
| SLC1A3 | solute carrier family 1 (glial high affinity glutamate transporter), member 3 | 2.243080993 | 6.06E-08 |
| GBP1 | guanylate binding protein 1, interferon-inducible | 2.241110771 | 0.000165394 |
| C1orf198 | chromosome 1 open reading frame 198 | 2.223852368 | 1.26E-07 |
| NA | NA | 2.21641348 | 0.002011135 |
| DHRS3 | dehydrogenase/reductase (SDR family) member 3 | 2.205868216 | 1.57E-06 |
| TSPAN9 | tetraspanin 9 | 2.203100526 | 4.39E-07 |
| PITPNC1 | phosphatidylinositol transfer protein, cytoplasmic 1 | 2.202311018 | 1.80E-05 |
| MLLT11 | myeloid/lymphoid or mixed-lineage leukemia (trithorax homolog, Drosophila); translocated to, 11 | 2.200318638 | 6.71E-08 |
| GABARAPL1 | GABA(A) receptor-associated protein like 1 | 2.196985622 | 6.22E-07 |
| TGM2 | transglutaminase 2 (C polypeptide, protein-glutamine-gamma-glutamyltransferase) | 2.187299325 | 5.12E-07 |
| LMO4 | LIM domain only 4 | 2.186174256 | 6.68E-07 |
| RAB31 | RAB31, member RAS oncogene family | 2.184067443 | 1.62E-08 |
| DHX58 | DEXH (Asp-Glu-X-His) box polypeptide 58 | 2.18238707 | 8.33E-09 |
| BDKRB1 | bradykinin receptor B1 | 2.168049512 | 5.91E-06 |
| CH25H | cholesterol 25-hydroxylase | 2.165768445 | 1.14E-06 |
| GPNMB | glycoprotein (transmembrane) nmb | 2.163697967 | 1.41E-07 |
| ITGA1 | integrin, alpha 1 | 2.157110148 | 2.38E-07 |
| NA | NA | 2.155925146 | 1.57E-06 |
| CNIH3 | cornichon homolog 3 (Drosophila) | 2.153018924 | 4.21E-07 |
| TGFBI | transforming growth factor, beta-induced, 68kDa | 2.152662609 | 3.87E-10 |
| VEGFA | vascular endothelial growth factor A | 2.134847877 | 0.001237142 |
| PSMB8 | proteasome (prosome, macropain) subunit, beta type, 8 (large multifunctional peptidase 7) | 2.128462908 | 8.70E-09 |
| TRIB3 | tribbles homolog 3 (Drosophila) | 2.105042363 | 8.86E-08 |
| TOM1 | target of myb1 (chicken) | 2.098391609 | 1.79E-05 |
| ITPRIP | inositol 1,4,5-trisphosphate receptor interacting protein | 2.09549626 | 1.18E-08 |
| GFPT2 | glutamine-fructose-6-phosphate transaminase 2 | 2.083398753 | 2.31E-06 |
| CASP1 | caspase 1, apoptosis-related cysteine peptidase (interleukin 1, beta, convertase) | 2.077112253 | 1.33E-08 |
| OAS2 | 2'-5'-oligoadenylate synthetase 2, 69/71kDa | 2.059104611 | 2.57E-09 |
| USP18 | ubiquitin specific peptidase 18 | 2.054785956 | 2.63E-07 |
| H1F0 | H1 histone family, member 0 | 2.054294994 | 5.66E-06 |
| NA | NA | 2.051311778 | 4.95E-07 |
| PARP14 | poly (ADP-ribose) polymerase family, member 14 | 2.047155591 | 3.42E-08 |
| SH3KBP1 | SH3-domain kinase binding protein 1 | 2.045431532 | 4.56E-09 |
| EREG | epiregulin | 2.041837221 | 0.001028117 |
| KLF4 | Kruppel-like factor 4 (gut) | 2.040464529 | 2.14E-07 |
| LAP3 | leucine aminopeptidase 3 | 2.040069812 | 8.03E-06 |
| TAPBP | TAP binding protein (tapasin) | 2.036137808 | 0.000124863 |
| CXCL10 | chemokine (C-X-C motif) ligand 10 | 2.030591081 | 2.30E-05 |
| NINJ1 | ninjurin 1 | 2.028914395 | 5.72E-09 |
| NAB1 | NGFI-A binding protein 1 (EGR1 binding protein 1) | 2.028157554 | 1.94E-06 |
| PHLDA1 | pleckstrin homology-like domain, family A, member 1 | 2.022546918 | 1.11E-08 |
| CSF3 | colony stimulating factor 3 (granulocyte) | 2.021078129 | 4.44E-05 |
| S100A3 | S100 calcium binding protein A3 | 2.017789742 | 8.95E-09 |
| MMP1 | matrix metallopeptidase 1 (interstitial collagenase) | 2.015078979 | 4.31E-08 |
| CSRNP1 | cysteine-serine-rich nuclear protein 1 | 2.014588405 | 2.47E-07 |
| CST3 | cystatin C | 2.013671547 | 0.000124772 |
| P4HA2 | prolyl 4-hydroxylase, alpha polypeptide II | 2.008038687 | 4.24E-08 |
| PTPRE | protein tyrosine phosphatase, receptor type, E | 2.006792158 | 9.59E-09 |
| BEX1 | brain expressed, X-linked 1 | 2.006346019 | 1.78E-07 |

**Supplementary file 1B.** Fold change (FC) for significantly altered genes (p-value <0.05) in YUGASP cells expressing IFI6 shRNAs

| **Gene Symbol** | **IFI6 shRNA1-FC** | **IFI6 shRNA2-FC** |
| --- | --- | --- |
| ACTN1 | 1.271452905 | 1.417344872 |
| AIF1L | -1.516322554 | -2.033283641 |
| APOD | 1.31378695 | 1.390322653 |
| ATAD2 | 1.368939895 | 1.759270334 |
| AURKB | 1.315546116 | 1.823782554 |
| AXL | -1.581589543 | -2.034777006 |
| AXL | -1.403574981 | -2.034777006 |
| BASP1 | -1.493582052 | -1.752518258 |
| BEX1 | -1.435694154 | -1.906364109 |
| BMP1 | -1.294384848 | -1.461615912 |
| C3orf14 | 1.256108711 | 1.209421276 |
| CACYBP | 1.382579702 | 1.585639245 |
| CADM1 | -1.271767955 | -1.241269924 |
| CAMK2N1 | -1.475431939 | -1.700904745 |
| CCDC77 | 1.297487962 | 1.548502526 |
| CCNE1 | 1.300578858 | 1.228757046 |
| CCNF | 1.312174298 | 2.024078474 |
| CD96 | -1.323918954 | -1.686778851 |
| CDC45 | 1.562031833 | 1.940822293 |
| CDCA8 | 1.247917805 | 2.031554004 |
| CDH19 | 1.272350421 | 1.210814243 |
| CDK1 | 1.307937652 | 1.923031258 |
| CELSR3 | -1.288869224 | -1.366423726 |
| CENPA | 1.321630988 | 1.551939427 |
| CENPM | 1.252944668 | 1.401590987 |
| CENPM | 1.328842187 | 1.401590987 |
| CKS1B | 1.318851755 | 1.899909757 |
| CKS1B | 1.325167012 | 1.899909757 |
| CNTNAP1 | -1.213126947 | -1.364732309 |
| COL18A1 | -1.339193091 | -1.554779516 |
| COL5A1 | -2.071417025 | -2.996380342 |
| COX5B | -1.26899404 | -1.20316785 |
| CPE | -1.256996878 | -1.219094883 |
| CPN1 | 1.548667263 | 1.586995043 |
| CTGF | 1.707580716 | 1.475902643 |
| CTNNA1 | -1.259562172 | -1.370283107 |
| CTSD | -1.398906501 | -1.614950427 |
| DEGS1 | 1.270890397 | 1.267042164 |
| DENND2A | -1.246737603 | -1.295365718 |
| DKK3 | -1.33941371 | -1.907730945 |
| DNER | -1.739256967 | -2.492711591 |
| DONSON | 1.300362552 | 1.279745662 |
| DPYSL2 | -1.479811352 | -1.721856199 |
| DSN1 | 1.289664422 | 1.481417727 |
| DTL | 1.310885428 | 1.728376892 |
| E2F2 | 1.544190365 | 2.885657659 |
| EFEMP1 | -1.475539912 | -2.134742374 |
| ELP2 | -1.302434767 | -1.346894585 |
| ENG | -1.267088355 | -1.487209408 |
| EPB41L4A-AS1 | -1.391135725 | -1.332383489 |
| EXO1 | 1.495963085 | 1.540139115 |
| FAM111A | 1.251570638 | 1.446597144 |
| FAM20C | -1.331550601 | -1.404672934 |
| FAM9C | 1.24633525 | 1.170882379 |
| FANCD2 | 1.315223936 | 1.160333517 |
| FBXO5 | 1.354450709 | 1.821607344 |
| FEN1 | 1.353543563 | 1.787365933 |
| FGFRL1 | -1.362568468 | -1.498665717 |
| FOXD1 | -1.277786984 | -1.367722376 |
| FTH1P2 | -1.248837354 | -1.360974494 |
| GANAB | -1.38783823 | -1.316133757 |
| GAS6 | -1.729310231 | -1.703233225 |
| GAS6 | -1.514334674 | -1.703233225 |
| GINS2 | 1.456730119 | 2.219351413 |
| GINS3 | 1.252556909 | 1.685487162 |
| HAT1 | 1.32064789 | 1.332373575 |
| HIPK2 | -1.370496143 | -1.420847951 |
| HIST2H2AA3 | -1.29404121 | -1.301812349 |
| HLA-DRA | 1.445658936 | 1.215735343 |
| HLA-DRA | 1.537965539 | 1.215735343 |
| HLA-DRB5 | 2.077842193 | 1.835244439 |
| HMGB2 | 1.263142325 | 1.515908449 |
| IARS2 | 1.33872799 | 1.273364769 |
| IFI6 | -2.259142326 | -2.209243478 |
| IFI6 | -2.158939505 | -2.209243478 |
| IGFBP5 | -1.889635866 | -2.161953322 |
| IGFBP5 | -1.848241443 | -2.161953322 |
| IL13RA1 | -1.216511382 | -1.316566872 |
| IL13RA2 | -1.495111171 | -1.44441846 |
| IL1B | -1.352712223 | -1.395742685 |
| IRF2BP2 | -1.252382863 | -1.413165463 |
| KIAA0101 | 1.543844374 | 2.132734385 |
| KIF20B | 1.313747384 | 2.029042856 |
| KIF4A | 1.228204972 | 1.616515577 |
| KLF6 | -1.341460403 | -1.357343527 |
| KLRC3 | -1.312261307 | -1.362140087 |
| KPNA2 | 1.3962806 | 1.378623321 |
| LAMA5 | -1.436467744 | -1.461027978 |
| LARP6 | -1.362204533 | -1.750906997 |
| LARP6 | -1.24409055 | -1.750906997 |
| LDLR | 1.428669586 | 1.967245854 |
| LDOC1 | -1.313588651 | -1.370763724 |
| LEMD1 | -1.342357307 | -1.706182519 |
| LEPREL1 | -1.293112466 | -1.489911996 |
| LOXL4 | 1.635628293 | 1.698522276 |
| LRRC8A | -1.256824095 | -1.395536996 |
| MCM10 | 1.303695011 | 1.530178613 |
| MCM3 | 1.320640611 | 1.5868974 |
| MCM6 | 1.33002283 | 1.794018362 |
| MECOM | -1.23440234 | -1.19974714 |
| MELK | 1.395888847 | 1.778641006 |
| MELK | 1.460999132 | 1.778641006 |
| MGST1 | -1.285743511 | 1.26222212 |
| MNS1 | 1.333670675 | 1.283772379 |
| MRAP2 | 1.26241702 | 1.216646443 |
| MSH6 | 1.301523232 | 1.465735314 |
| MSX1 | -1.276680099 | -1.266561492 |
| MXD4 | -1.425757155 | -2.084164434 |
| MXRA7 | -1.246714716 | -1.419667019 |
| NAV2 | -1.22188793 | -1.549198017 |
| NCAPG2 | 1.391313868 | 1.814317859 |
| NREP | -1.441642353 | -1.854218316 |
| NUAK1 | -1.229604992 | -1.275177895 |
| NUSAP1 | 1.359525299 | 1.863742833 |
| OLFML2B | -1.353191044 | -1.677203484 |
| ORC6 | 1.282003612 | 1.747646341 |
| PINK1 | -1.268321236 | -1.470834646 |
| PKMYT1 | 1.280701854 | 1.202928412 |
| PLK4 | 1.238761814 | 1.827582613 |
| PLSCR3 | -1.267447048 | -1.427138839 |
| PMEL | -1.31182604 | 1.57599896 |
| PMEPA1 | -2.027495236 | -2.976908999 |
| PMEPA1 | -1.537375284 | -2.976908999 |
| PODXL | -1.299217413 | -1.425238804 |
| POLA1 | 1.30158141 | 1.514444247 |
| POLA2 | 1.367000744 | 1.620720031 |
| PPIC | -1.301778368 | -1.960240413 |
| PRADC1 | 1.20633761 | 1.231198668 |
| PRIM1 | 1.344312215 | 1.683398209 |
| PRR14L | -1.23114576 | -1.238741015 |
| PTPRF | -1.477756473 | -1.801736693 |
| PTPRR | -1.452129785 | -1.281828891 |
| RAD51AP1 | 1.503491231 | 2.003881284 |
| RAD51C | 1.318436114 | 1.334626429 |
| RAD54L | 1.281920883 | 1.479671649 |
| RFC4 | 1.59358582 | 1.795312128 |
| RGS1 | 1.497520769 | 1.405221874 |
| RHBDF2 | -1.309500704 | -1.480745502 |
| RNASET2 | -1.552211993 | -1.48698645 |
| RRM2 | 1.385554055 | 2.177947005 |
| SCG2 | -1.705930092 | -2.435555356 |
| SEPP1 | -1.656295745 | -2.02227674 |
| SERPINA3 | 1.700314832 | 1.607979646 |
| SH3BP4 | -1.337821998 | -1.5125277 |
| SLBP | 1.304116452 | 1.394395655 |
| SLC35F2 | -1.32423898 | -1.321343797 |
| SLCO4A1-AS1 | -1.469297892 | -1.78208251 |
| SMC4 | 1.327340994 | 1.238711557 |
| SNRNP70 | 1.345746839 | 1.481284385 |
| SOX2 | -1.246506661 | -1.343225837 |
| SPANXA1 | -1.577238693 | -1.793795747 |
| SPANXA2 | -2.213321385 | -2.592293023 |
| SPANXB1 | -2.061142773 | -2.322064752 |
| SPANXC | -1.718487141 | -1.802787164 |
| SPC25 | 1.291339084 | 1.631437756 |
| SPOCK1 | -1.505049934 | -2.012702112 |
| SPRY4 | 1.218027065 | 1.162749215 |
| SPRY4 | 1.225261757 | 1.162749215 |
| ST6GALNAC2 | -1.406636601 | -1.779397399 |
| STC1 | -1.622919302 | -1.759668046 |
| STMN3 | -1.310161227 | -1.255097771 |
| STX16 | -1.416709659 | -1.222300603 |
| TBL1X | -1.290550874 | -1.387852556 |
| TCN1 | -1.418392121 | -1.827596803 |
| TF | -1.486671745 | -1.810759237 |
| TMEM97 | 1.327345454 | -1.278101797 |
| TMSB4X | 1.801388147 | 1.422021564 |
| TMTC1 | -1.238475479 | -1.278739714 |
| TNFRSF12A | 1.309853429 | 1.634042964 |
| TOX2 | -1.400120934 | -1.429670011 |
| TRIM51 | 1.543689717 | 1.829683974 |
| TRIM51EP | 1.298004214 | 1.424438081 |
| TUBB2B | -1.40310168 | -1.608767578 |
| TYMS | 1.574898564 | 2.250906124 |
| UBE2T | 1.309782947 | 1.877670395 |
| UHRF1 | 1.517955282 | 1.216736186 |
| VCX | -1.687630546 | -1.972914625 |
| VCX3A | -1.441982027 | -1.683406042 |
| VCX3B | -1.384918758 | -1.870000532 |
| ZWILCH | 1.391772115 | 1.240216531 |
| ZWINT | 1.321018139 | 1.567996588 |

Supplementary file 1C. Biological Pathway Enrichment Analysis report

Enrichment by Pathway Maps YUGASP-common

# Maps Total pValue Min FDR p-value FDR In Data "Network Objects from

Active Data"

1 Cell cycle_The metaphase checkpoint 36 4.464E-07 9.598E-05 4.464E-07 9.598E-05 6 "DSN1, Zwilch, Aurora-B, SPBC25, CENP-A, HZwint-1"

2 Cell cycle_Start of DNA replication in early S phase 32 6.037E-06 6.490E-04 6.037E-06 6.490E-04 5 "MCM3, MCM10, ORC6L, Cyclin E, CDC45L"

3 Cell cycle_Chromosome condensation in prometaphase 21 2.423E-05 1.737E-03 2.423E-05 1.737E-03 4 "Aurora-B, CAP-C, CAP-G/G2, CDK1 (p34)"

4 Cell cycle_Role of SCF complex in cell cycle regulation 29 9.142E-05 4.914E-03 9.142E-05 4.914E-03 4 "Emi1, Cyclin E, CDK1 (p34), CKS1"

5 Cell cycle_Role of APC in cell cycle regulation 32 1.358E-04 5.840E-03 1.358E-04 5.840E-03 4 "Aurora-B, Emi1, CDK1 (p34), CKS1"

6 Cell cycle_Nucleocytoplasmic transport of CDK/Cyclins 14 1.912E-04 6.853E-03 1.912E-04 6.853E-03 3 "Importin (karyopherin)-alpha, Cyclin E, CDK1 (p34)"

7 Cell cycle_Cell cycle (generic schema) 21 6.699E-04 2.058E-02 6.699E-04 2.058E-02 3 "E2F2, Cyclin E, CDK1 (p34)"

8 DNA damage_ATM / ATR regulation of G2 / M checkpoint 26 1.271E-03 3.416E-02 1.271E-03 3.416E-02 3 "Kinase MYT1, FANCD2, CDK1 (p34)"

9 Transcription_Ligand-dependent activation of the ESR1/SP pathway 30 1.938E-03 4.629E-02 1.938E-03 4.629E-02 3 "TYSY, Cyclin E, LDLR"

10 Cell cycle_Spindle assembly and chromosome separation 33 2.557E-03 5.498E-02 2.557E-03 5.498E-02 3 "Aurora-B, Importin (karyopherin)-alpha, CDK1 (p34)"

11 dCTP/dUTP metabolism 75 3.501E-03 6.842E-02 3.501E-03 6.842E-02 4 "RRM2, POLA1, Small RR subunit, POLA2"

12 Development_BMP7 in brown adipocyte differentiation 39 4.133E-03 7.129E-02 4.133E-03 7.129E-02 3 "COX Vb, SERPINA3 (ACT), COL5A1"

13 Immune response_Antigen presentation by MHC class II 12 4.310E-03 7.129E-02 4.310E-03 7.129E-02 2 "HLA-DRA1, MHC class II"

14 High shear stress-induced platelet activation 46 6.584E-03 1.011E-01 6.584E-03 1.011E-01 3 "Alpha-actinin, UFO, Gas6"

15 dATP/dITP metabolism 95 8.089E-03 1.159E-01 8.089E-03 1.159E-01 4 "RRM2, POLA1, Small RR subunit, POLA2"

16 Immune response_HMGB1/RAGE signaling pathway 53 9.746E-03 1.310E-01 9.746E-03 1.310E-01 3 "Tissue factor, IL-1 beta, Secretogranin II"

17 Aberrant B-Raf signaling in melanoma progression 55 1.079E-02 1.364E-01 1.079E-02 1.364E-01 3 "Aurora-B, Kinase MYT1, CDK1 (p34)"

18 DNA damage_Mismatch repair 20 1.188E-02 1.420E-01 1.188E-02 1.420E-01 2 "EXO1, MSH6"

19 Expression targets of Tissue factor signaling in cancer 22 1.429E-02 1.617E-01 1.429E-02 1.617E-01 2 "Tissue factor, CTGF"

20 Cell adhesion_Endothelial cell contacts by non-junctional mechanisms 24 1.690E-02 1.693E-01 1.690E-02 1.693E-01 2 "Alpha-actinin, Alpha-catenin"

21 Proteolysis_Role of Parkin in the Ubiquitin-Proteasomal Pathway 24 1.690E-02 1.693E-01 1.690E-02 1.693E-01 2 "Tubulin beta, Cyclin E"

22 TTP metabolism 66 1.762E-02 1.693E-01 1.762E-02 1.693E-01 3 "POLA1, TYSY, POLA2"

23 Cell cycle_Initiation of mitosis 25 1.827E-02 1.693E-01 1.827E-02 1.693E-01 2 "Kinase MYT1, CDK1 (p34)"

24 Cell adhesion_Cadherin-mediated cell adhesion 26 1.968E-02 1.693E-01 1.968E-02 1.693E-01 2 "PTPRF (LAR), Alpha-catenin"

25 Cell adhesion_Endothelial cell contacts by junctional mechanisms 26 1.968E-02 1.693E-01 1.968E-02 1.693E-01 2 "Alpha-actinin, Alpha-catenin"

26 Immune response_Role of HMGB1 in dendritic cell maturation and migration 27 2.114E-02 1.748E-01 2.114E-02 1.748E-01 2 "IL-1 beta, MHC class II"

27 Cell cycle_Transition and termination of DNA replication 28 2.265E-02 1.804E-01 2.265E-02 1.804E-01 2 "FEN1, CDK1 (p34)"

28 DNA damage_Role of Brca1 and Brca2 in DNA repair 30 2.579E-02 1.980E-01 2.579E-02 1.980E-01 2 "MSH6, FANCD2"

29 Cortisol biosynthesis from Cholesterol 31 2.742E-02 2.018E-01 2.742E-02 2.018E-01 2 "CYP11B2, CYP11B1"

30 DNA damage_ATM/ATR regulation of G1/S checkpoint 32 2.909E-02 2.018E-01 2.909E-02 2.018E-01 2 "FANCD2, Cyclin E"

31 Cell cycle_Role of Nek in cell cycle regulation 32 2.909E-02 2.018E-01 2.909E-02 2.018E-01 2 "Tubulin beta, CDK1 (p34)"

32 Cell cycle_ESR1 regulation of G1/S transition 33 3.080E-02 2.069E-01 3.080E-02 2.069E-01 2 "Cyclin E, CKS1"

33 Immune response_Th17 cell differentiation 35 3.434E-02 2.237E-01 3.434E-02 2.237E-01 2 "IL-1 beta, MHC class II"

34 Cytoskeleton remodeling_Keratin filaments 36 3.617E-02 2.287E-01 3.617E-02 2.287E-01 2 "Tubulin beta, CDK1 (p34)"

35 Substance P-mediated inflammation and pain in Sickle cell disease 38 3.993E-02 2.453E-01 3.993E-02 2.453E-01 2 "Tissue factor, IL-1 beta"

36 "Immune response_Th17, Th22 and Th9 cell differentiation " 39 4.187E-02 2.480E-01 4.187E-02 2.480E-01 2 "IL-1 beta, MHC class II"

37 Transcription_Role of heterochromatin protein 1 (HP1) family in transcriptional silencing 40 4.384E-02 2.480E-01 4.384E-02 2.480E-01 2 "Cyclin E, CDK1 (p34)"

38 Reproduction_Progesterone-mediated oocyte maturation 40 4.384E-02 2.480E-01 4.384E-02 2.480E-01 2 "Kinase MYT1, CDK1 (p34)"

39 Androstenedione and testosterone biosynthesis and metabolism p.3 41 4.584E-02 2.527E-01 4.584E-02 2.527E-01 2 "CYP11B2, CYP11B1"

40 Apoptosis and survival_Anti-apoptotic TNFs/NF-kB/Bcl-2 pathway 42 4.788E-02 2.543E-01 4.788E-02 2.543E-01 2 "BAFF(TNFSF13B), FN14(TNFRSF12A)"

41 Role of platelets in allograft rejection 43 4.995E-02 2.543E-01 4.995E-02 2.543E-01 2 "Tissue factor, IL-1 beta"

42 Immune response_IL-13 signaling via JAK-STAT 44 5.205E-02 2.543E-01 5.205E-02 2.543E-01 2 "IL13RA2, IL13RA1"

43 Immune response_IL-1 signaling pathway 44 5.205E-02 2.543E-01 5.205E-02 2.543E-01 2 "Tissue factor, IL-1 beta"

44 Androstenedione and testosterone biosynthesis and metabolism p.3/ Rodent version 44 5.205E-02 2.543E-01 5.205E-02 2.543E-01 2 "CYP11B2, CYP11B1"

45 Cell adhesion_Histamine H1 receptor signaling in the interruption of cell barrier integrity 45 5.418E-02 2.577E-01 5.418E-02 2.577E-01 2 "Alpha-actinin, Alpha-catenin"

46 Immune response_Naive CD4+ T cell differentiation 46 5.635E-02 2.577E-01 5.635E-02 2.577E-01 2 "IL-1 beta, MHC class II"

47 Immune response_Inhibitory action of Lipoxins on pro-inflammatory TNF-alpha signaling 46 5.635E-02 2.577E-01 5.635E-02 2.577E-01 2 "IL-1 beta, Cyclin E"

48 Immune response_NF-AT signaling and leukocyte interactions 47 5.854E-02 2.613E-01 5.854E-02 2.613E-01 2 "IL13RA1, MHC class II"

49 IL-17-induced mucin expression in CF airways 48 6.076E-02 2.613E-01 6.076E-02 2.613E-01 2 "IL-1 beta, MHC class II"

50 Immune response_C3a signaling 48 6.076E-02 2.613E-01 6.076E-02 2.613E-01 2 "IL-1 beta, MHC class II"

51 Th17 cells in CF (mouse model) 49 6.301E-02 2.632E-01 6.301E-02 2.632E-01 2 "IL-1 beta, MHC class II"

52 Immune responses in asthma (schema) 8 6.496E-02 2.632E-01 6.496E-02 2.632E-01 1 IL-1 beta

53 Cortisone biosynthesis and metabolism 50 6.529E-02 2.632E-01 6.529E-02 2.632E-01 2 "CYP11B2, CYP11B1"

54 Signal transduction_NF-kB activation pathways 51 6.760E-02 2.632E-01 6.760E-02 2.632E-01 2 "IL-1 beta, BAFF(TNFSF13B)"

55 Immune response_Inhibitory PD-1 signaling in T cells 53 7.229E-02 2.632E-01 7.229E-02 2.632E-01 2 "Cyclin E, MHC class II"

56 Cell adhesion_Role of CDK5 in cell adhesion 9 7.278E-02 2.632E-01 7.278E-02 2.632E-01 1 Alpha-catenin

57 Hypoxia-induced EMT in cancer and fibrosis 9 7.278E-02 2.632E-01 7.278E-02 2.632E-01 1 CTGF

58 Immune response_HSP60 and HSP70/ TLR signaling pathway 54 7.468E-02 2.632E-01 7.468E-02 2.632E-01 2 "IL-1 beta, MHC class II"

59 Immune response_CCL2 signaling 54 7.468E-02 2.632E-01 7.468E-02 2.632E-01 2 "IL-1 beta, CDH19"

60 Th17 cells in CF 54 7.468E-02 2.632E-01 7.468E-02 2.632E-01 2 "IL-1 beta, MHC class II"

61 Neurophysiological process_Dynein-dynactin motor complex in axonal transport in neurons 54 7.468E-02 2.632E-01 7.468E-02 2.632E-01 2 "Carboxypeptidase H, Importin (karyopherin)-alpha"

62 Immune response_Role of PKR in stress-induced antiviral cell response 57 8.199E-02 2.843E-01 8.199E-02 2.843E-01 2 "IL-1 beta, BAFF(TNFSF13B)"

63 Immune response_Immunological synapse formation 59 8.698E-02 2.968E-01 8.698E-02 2.968E-01 2 "Alpha-actinin, MHC class II"

64 Immune response_IL-10 signaling pathway 62 9.463E-02 3.179E-01 9.463E-02 3.179E-01 2 "IL-1 beta, MHC class II"

65 dGTP metabolism 65 1.025E-01 3.389E-01 1.025E-01 3.389E-01 2 "POLA1, POLA2"

66 Type 2 diabetes (general schema) 14 1.109E-01 3.614E-01 1.109E-01 3.614E-01 1 IL-1 beta

67 Inhibition of neutrophil migration by proresolving lipid mediators in COPD 70 1.159E-01 3.644E-01 1.159E-01 3.644E-01 2 "Alpha-actinin, IL-1 beta"

68 Apoptosis and survival_DNA-damage-induced apoptosis 15 1.184E-01 3.644E-01 1.184E-01 3.644E-01 1 FANCD2

69 Metabolic syndrome X (general schema) 15 1.184E-01 3.644E-01 1.184E-01 3.644E-01 1 IL-1 beta

70 Blood coagulation_Platelet microparticle generation 71 1.186E-01 3.644E-01 1.186E-01 3.644E-01 2 "Tissue factor, IL-1 beta"

71 Immune response_Oncostatin M signaling via JAK-Stat in mouse cells 18 1.403E-01 4.191E-01 1.403E-01 4.191E-01 1 SERPINA3 (ACT)

72 Transport_RAN regulation pathway 18 1.403E-01 4.191E-01 1.403E-01 4.191E-01 1 Importin (karyopherin)-alpha

73 DNA damage_NHEJ mechanisms of DSBs repair 19 1.475E-01 4.263E-01 1.475E-01 4.263E-01 1 FEN1

74 Protein folding and maturation_Insulin processing 20 1.547E-01 4.263E-01 1.547E-01 4.263E-01 1 Carboxypeptidase H

75 Immune response_Oncostatin M signaling via JAK-Stat in human cells 20 1.547E-01 4.263E-01 1.547E-01 4.263E-01 1 SERPINA3 (ACT)

76 Development_FGF2-dependent induction of EMT 20 1.547E-01 4.263E-01 1.547E-01 4.263E-01 1 IL-1 beta

77 Cell cycle_Sister chromatid cohesion 22 1.688E-01 4.263E-01 1.688E-01 4.263E-01 1 CDK1 (p34)

78 Cell cycle_Role of 14-3-3 proteins in cell cycle regulation 22 1.688E-01 4.263E-01 1.688E-01 4.263E-01 1 CDK1 (p34)

79 Development_Thrombopoetin signaling via JAK-STAT pathway 22 1.688E-01 4.263E-01 1.688E-01 4.263E-01 1 SERPINA3 (ACT)

80 LRRK2 and immune function in Parkinson's disease 22 1.688E-01 4.263E-01 1.688E-01 4.263E-01 1 MHC class II

81 Immune response_MIF-JAB1 signaling 24 1.826E-01 4.263E-01 1.826E-01 4.263E-01 1 Cyclin E

82 Immune response_IFN alpha/beta signaling pathway 24 1.826E-01 4.263E-01 1.826E-01 4.263E-01 1 IFI6

83 CFTR folding and maturation (normal and CF) 24 1.826E-01 4.263E-01 1.826E-01 4.263E-01 1 "Glucosidase II, alpha subunits"

84 Immune response_LPS-induced platelet activation 25 1.895E-01 4.263E-01 1.895E-01 4.263E-01 1 IL-1 beta

85 Cytoskeleton remodeling_Neurofilaments 25 1.895E-01 4.263E-01 1.895E-01 4.263E-01 1 Tubulin beta

86 Cell cycle_Regulation of G1/S transition (part 2) 26 1.963E-01 4.263E-01 1.963E-01 4.263E-01 1 Cyclin E

87 Transcription_Role of Akt in hypoxia induced HIF1 activation 27 2.030E-01 4.263E-01 2.030E-01 4.263E-01 1 Transferrin

88 HCV-dependent regulation of membrane receptors signaling in HCC 27 2.030E-01 4.263E-01 2.030E-01 4.263E-01 1 IL-1 beta

89 Cell adhesion_Chemokines and adhesion 100 2.035E-01 4.263E-01 2.035E-01 4.263E-01 2 "Alpha-actinin, Alpha-actinin 1"

90 Cytoskeleton remodeling_Cytoskeleton remodeling 102 2.096E-01 4.263E-01 2.096E-01 4.263E-01 2 "Alpha-actinin, Alpha-actinin 1"

91 Mitogenic action of Estradiol / ESR1 (nuclear) in breast cancer 28 2.097E-01 4.263E-01 2.097E-01 4.263E-01 1 Cyclin E

92 Immune response_CD137 signaling in immune cell 29 2.163E-01 4.263E-01 2.163E-01 4.263E-01 1 Cyclin E

93 Apoptosis and survival_p53-dependent apoptosis 29 2.163E-01 4.263E-01 2.163E-01 4.263E-01 1 CDK1 (p34)

94 Cholesterol and Sphingolipid transport / Influx to the early endosome in lung (normal and CF) 29 2.163E-01 4.263E-01 2.163E-01 4.263E-01 1 LDLR

95 Heme metabolism 105 2.188E-01 4.263E-01 2.188E-01 4.263E-01 2 "Holotransferrin, Apotransferrin"

96 Protein folding and maturation_POMC processing 30 2.229E-01 4.263E-01 2.229E-01 4.263E-01 1 Carboxypeptidase H

97 Apoptosis and survival_Granzyme A signaling 30 2.229E-01 4.263E-01 2.229E-01 4.263E-01 1 HMG2

98 Development_Slit-Robo signaling 30 2.229E-01 4.263E-01 2.229E-01 4.263E-01 1 CRMP2

99 Cell adhesion_Gap junctions 30 2.229E-01 4.263E-01 2.229E-01 4.263E-01 1 Tubulin beta

100 Cytoskeleton remodeling_Fibronectin-binding integrins in cell motility 31 2.294E-01 4.263E-01 2.294E-01 4.263E-01 1 Alpha-actinin

101 Apoptosis and survival_Role of IAP-proteins in apoptosis 31 2.294E-01 4.263E-01 2.294E-01 4.263E-01 1 CDK1 (p34)

102 IL-1 beta-dependent CFTR expression 31 2.294E-01 4.263E-01 2.294E-01 4.263E-01 1 IL-1 beta

103 Protein folding and maturation_Bradykinin / Kallidin maturation 32 2.359E-01 4.263E-01 2.359E-01 4.263E-01 1 Carboxypeptidase N (cat)

104 "Cytoskeleton remodeling_TGF, WNT and cytoskeletal remodeling" 111 2.374E-01 4.263E-01 2.374E-01 4.263E-01 2 "Alpha-actinin, Alpha-actinin 1"

105 G-protein signaling_N-RAS regulation pathway 33 2.423E-01 4.263E-01 2.423E-01 4.263E-01 1 MHC class II

106 Signal transduction_Activin A signaling regulation 33 2.423E-01 4.263E-01 2.423E-01 4.263E-01 1 Evi-1

107 Immune response_ETV3 affect on CSF1-promoted macrophage differentiation 33 2.423E-01 4.263E-01 2.423E-01 4.263E-01 1 CDK1 (p34)

108 Immune response_IL-22 signaling pathway 34 2.486E-01 4.263E-01 2.486E-01 4.263E-01 1 MHC class II

109 Immune response_Inflammasome in inflammatory response 34 2.486E-01 4.263E-01 2.486E-01 4.263E-01 1 IL-1 beta

110 Development_Role of cell-cell and ECM-cell interactions in oligodendrocyte differentiation and myelination 34 2.486E-01 4.263E-01 2.486E-01 4.263E-01 1 Caspr1

111 Signal transduction_PTMs in BAFF-induced non-canonical NF-kB signaling 34 2.486E-01 4.263E-01 2.486E-01 4.263E-01 1 BAFF(TNFSF13B)

112 Signal transduction_Erk Interactions: Inhibition of Erk 34 2.486E-01 4.263E-01 2.486E-01 4.263E-01 1 PTPRR

113 Apoptosis and survival_Role of CDK5 in neuronal death and survival 34 2.486E-01 4.263E-01 2.486E-01 4.263E-01 1 CDK1 (p34)

114 Role of Tissue factor in cancer independent of coagulation protease signaling 35 2.549E-01 4.263E-01 2.549E-01 4.263E-01 1 Tissue factor

115 Immune response_Differentiation of natural regulatory T cells 35 2.549E-01 4.263E-01 2.549E-01 4.263E-01 1 MHC class II

116 Immune response_Oncostatin M signaling via MAPK in mouse cells 35 2.549E-01 4.263E-01 2.549E-01 4.263E-01 1 LDLR

117 Upregulation of MITF in melanoma 36 2.612E-01 4.263E-01 2.612E-01 4.263E-01 1 PM17

118 Development_SSTR2 in regulation of cell proliferation 36 2.612E-01 4.263E-01 2.612E-01 4.263E-01 1 Cyclin E

119 Immune response_HMGB1/TLR signaling pathway 36 2.612E-01 4.263E-01 2.612E-01 4.263E-01 1 IL-1 beta

120 Complement pathway disruption in thrombotic microangiopathy 37 2.674E-01 4.263E-01 2.674E-01 4.263E-01 1 IL-1 beta

121 Immune response_Oncostatin M signaling via MAPK in human cells 37 2.674E-01 4.263E-01 2.674E-01 4.263E-01 1 LDLR

122 Role of red blood cell adhesion to endothelium in vaso-occlusion in Sickle cell disease 37 2.674E-01 4.263E-01 2.674E-01 4.263E-01 1 LAMA5

123 Immune response_Generation of memory CD4+ T cells 37 2.674E-01 4.263E-01 2.674E-01 4.263E-01 1 MHC class II

124 Transcription_Role of AP-1 in regulation of cellular metabolism 38 2.735E-01 4.263E-01 2.735E-01 4.263E-01 1 POLA1

125 Aldosterone biosynthesis and metabolism 38 2.735E-01 4.263E-01 2.735E-01 4.263E-01 1 CYP11B2

126 "Regulation of lipid metabolism_Regulation of lipid metabolism via LXR, NF-Y and SREBP" 38 2.735E-01 4.263E-01 2.735E-01 4.263E-01 1 LDLR

127 Cell cycle_Regulation of G1/S transition (part 1) 38 2.735E-01 4.263E-01 2.735E-01 4.263E-01 1 Cyclin E

128 ATP/ITP metabolism 124 2.777E-01 4.263E-01 2.777E-01 4.263E-01 2 "RRM2, Small RR subunit"

129 Apoptosis and survival_APRIL and BAFF signaling 39 2.796E-01 4.263E-01 2.796E-01 4.263E-01 1 BAFF(TNFSF13B)

130 Immune response_Differentiation and clonal expansion of CD8+ T cells 39 2.796E-01 4.263E-01 2.796E-01 4.263E-01 1 MHC class II

131 Blood coagulation_Blood coagulation 39 2.796E-01 4.263E-01 2.796E-01 4.263E-01 1 Tissue factor

132 Immune response_MIF in innate immunity response 40 2.857E-01 4.263E-01 2.857E-01 4.263E-01 1 IL-1 beta

133 Immune response_TCR and CD28 co-stimulation in activation of NF-kB 40 2.857E-01 4.263E-01 2.857E-01 4.263E-01 1 MHC class II

134 Immune response_Th1 and Th2 cell differentiation 40 2.857E-01 4.263E-01 2.857E-01 4.263E-01 1 MHC class II

135 Apoptosis and survival_Ceramides signaling pathway 40 2.857E-01 4.263E-01 2.857E-01 4.263E-01 1 Cathepsin D

136 Translation_(L)-selenoaminoacids incorporation in proteins during translation 41 2.917E-01 4.263E-01 2.917E-01 4.263E-01 1 Selenoprotein P

137 TLR2-induced platelet activation 41 2.917E-01 4.263E-01 2.917E-01 4.263E-01 1 IL-1 beta

138 N-Glycan biosynthesis p2 41 2.917E-01 4.263E-01 2.917E-01 4.263E-01 1 "Glucosidase II, alpha subunits"

139 Immune response_HMGB1 release from the cell 41 2.917E-01 4.263E-01 2.917E-01 4.263E-01 1 IL-1 beta

140 Estrogen biosynthesis 42 2.976E-01 4.263E-01 2.976E-01 4.263E-01 1 CYP11B1

141 Apoptosis and survival_BAD phosphorylation 42 2.976E-01 4.263E-01 2.976E-01 4.263E-01 1 CDK1 (p34)

142 Impaired inhibitory action of lipoxins and Resolvin E1 on neutrophil functions in CF 43 3.035E-01 4.263E-01 3.035E-01 4.263E-01 1 IL-1 beta

143 Protein folding and maturation_Angiotensin system maturation \ Human version 43 3.035E-01 4.263E-01 3.035E-01 4.263E-01 1 Cathepsin D

144 Regulation of Tissue factor signaling in cancer 43 3.035E-01 4.263E-01 3.035E-01 4.263E-01 1 Tissue factor

145 Role of cell adhesion in vaso-occlusion in Sickle cell disease 43 3.035E-01 4.263E-01 3.035E-01 4.263E-01 1 IL-1 beta

146 Apoptosis and survival_TNF-alpha-induced Caspase-8 signaling 43 3.035E-01 4.263E-01 3.035E-01 4.263E-01 1 Cathepsin D

147 Role of platelets in the initiation of in-stent restenosis 43 3.035E-01 4.263E-01 3.035E-01 4.263E-01 1 Tissue factor

148 Signal transduction_PTMs in BAFF-induced canonical NF-kB signaling 43 3.035E-01 4.263E-01 3.035E-01 4.263E-01 1 BAFF(TNFSF13B)

149 Immune response_PGE2 in immune and neuroendocrine system interactions 44 3.094E-01 4.263E-01 3.094E-01 4.263E-01 1 IL-1 beta

150 Neurophysiological process_Receptor-mediated axon growth repulsion 45 3.152E-01 4.263E-01 3.152E-01 4.263E-01 1 CRMP2

151 Role of alpha-6/beta-4 integrins in carcinoma progression 45 3.152E-01 4.263E-01 3.152E-01 4.263E-01 1 MSP

152 Immune response_ICOS pathway in T-helper cell 46 3.209E-01 4.263E-01 3.209E-01 4.263E-01 1 MHC class II

153 Regulation of GSK3 beta in bipolar disorder 46 3.209E-01 4.263E-01 3.209E-01 4.263E-01 1 IL-1 beta

154 FGF signaling in pancreatic cancer 46 3.209E-01 4.263E-01 3.209E-01 4.263E-01 1 Alpha-catenin

155 Immune response_MIF - the neuroendocrine-macrophage connector 46 3.209E-01 4.263E-01 3.209E-01 4.263E-01 1 MHC class II

156 Stimulation of TGF-beta signaling in lung cancer 48 3.323E-01 4.263E-01 3.323E-01 4.263E-01 1 IL-1 beta

157 Immune response_Histamine H1 receptor signaling in immune response 48 3.323E-01 4.263E-01 3.323E-01 4.263E-01 1 Tissue factor

158 "Immune response_TLR5, TLR7, TLR8 and TLR9 signaling pathways" 48 3.323E-01 4.263E-01 3.323E-01 4.263E-01 1 IL-1 beta

159 Cell adhesion_Integrin-mediated cell adhesion and migration 48 3.323E-01 4.263E-01 3.323E-01 4.263E-01 1 Alpha-actinin

160 Protein folding and maturation_Angiotensin system maturation \ Rodent version 48 3.323E-01 4.263E-01 3.323E-01 4.263E-01 1 Cathepsin D

161 Transcription_N-CoR/ SMRT complex-mediated epigenetic gene silencing 49 3.379E-01 4.263E-01 3.379E-01 4.263E-01 1 TBL1X

162 Role and regulation of Prostaglandin E2 in gastric cancer 49 3.379E-01 4.263E-01 3.379E-01 4.263E-01 1 IL-1 beta

163 Cytoskeleton remodeling_Integrin outside-in signaling 49 3.379E-01 4.263E-01 3.379E-01 4.263E-01 1 Alpha-actinin

164 Immune response_Bacterial infections in normal airways 49 3.379E-01 4.263E-01 3.379E-01 4.263E-01 1 IL-1 beta

165 PDE4 regulation of cyto/chemokine expression in arthritis 49 3.379E-01 4.263E-01 3.379E-01 4.263E-01 1 IL-1 beta

166 Development_PEDF signaling 49 3.379E-01 4.263E-01 3.379E-01 4.263E-01 1 IL-1 beta

167 Tissue Factor signaling in cancer via PAR1 and PAR2 49 3.379E-01 4.263E-01 3.379E-01 4.263E-01 1 Tissue factor

168 Immune response_C5a signaling 50 3.435E-01 4.263E-01 3.435E-01 4.263E-01 1 IL-1 beta

169 Protein folding and maturation_Posttranslational processing of neuroendocrine peptides 50 3.435E-01 4.263E-01 3.435E-01 4.263E-01 1 Carboxypeptidase H

170 Immune response_Histamine signaling in dendritic cells 50 3.435E-01 4.263E-01 3.435E-01 4.263E-01 1 IL-1 beta

171 PDE4 regulation of cyto/chemokine expression in inflammatory skin diseases 50 3.435E-01 4.263E-01 3.435E-01 4.263E-01 1 IL-1 beta

172 Immune response_IL-13 signaling via PI3K-ERK 50 3.435E-01 4.263E-01 3.435E-01 4.263E-01 1 IL13RA1

173 Signal transduction_PTMs in BAFF-induced signaling 51 3.490E-01 4.263E-01 3.490E-01 4.263E-01 1 BAFF(TNFSF13B)

174 Chemotaxis_Inhibitory action of lipoxins on IL-8- and Leukotriene B4-induced neutrophil migration 51 3.490E-01 4.263E-01 3.490E-01 4.263E-01 1 Alpha-actinin

175 Inflammatory factors-induced expression of mucins in normal and asthmatic epithelium 51 3.490E-01 4.263E-01 3.490E-01 4.263E-01 1 IL-1 beta

176 Immune response_NFAT in immune response 51 3.490E-01 4.263E-01 3.490E-01 4.263E-01 1 MHC class II

177 Development_IGF-1 receptor signaling 52 3.544E-01 4.281E-01 3.544E-01 4.281E-01 1 IBP

178 Immune response_Antiviral actions of Interferons 52 3.544E-01 4.281E-01 3.544E-01 4.281E-01 1 MHC class II

179 Cell cycle_Influence of Ras and Rho proteins on G1/S Transition 53 3.599E-01 4.298E-01 3.599E-01 4.298E-01 1 Cyclin E

180 Immune response_T cell receptor signaling pathway 53 3.599E-01 4.298E-01 3.599E-01 4.298E-01 1 MHC class II

181 Immune response_Role of DAP12 receptors in NK cells 54 3.653E-01 4.315E-01 3.653E-01 4.315E-01 1 KLRC3

182 Immune response_IL-16 signaling pathway 54 3.653E-01 4.315E-01 3.653E-01 4.315E-01 1 IL-1 beta

183 Apoptosis and survival_NO synthesis and signaling 55 3.706E-01 4.330E-01 3.706E-01 4.330E-01 1 IL-1 beta

184 Immune response_Platelet activating factor/ PTAFR pathway signaling 55 3.706E-01 4.330E-01 3.706E-01 4.330E-01 1 IL-1 beta

185 Impaired inhibitory action of lipoxins on neutrophil migration in CF 56 3.759E-01 4.359E-01 3.759E-01 4.359E-01 1 Alpha-actinin

186 Development_Cytokine-mediated regulation of megakaryopoiesis 57 3.811E-01 4.359E-01 3.811E-01 4.359E-01 1 IL-1 beta

187 Immune response_TLR2 and TLR4 signaling pathways 57 3.811E-01 4.359E-01 3.811E-01 4.359E-01 1 IL-1 beta

188 Transcription_Epigenetic regulation of gene expression 57 3.811E-01 4.359E-01 3.811E-01 4.359E-01 1 HAT1

189 Development_Regulation of cytoskeleton proteins in oligodendrocyte differentiation and myelination 58 3.863E-01 4.395E-01 3.863E-01 4.395E-01 1 Tubulin beta

190 Immune response_TREM1 signaling pathway 59 3.915E-01 4.418E-01 3.915E-01 4.418E-01 1 IL-1 beta

191 IGF family signaling in colorectal cancer 60 3.966E-01 4.418E-01 3.966E-01 4.418E-01 1 IBP

192 Immune response_IL-18 signaling 60 3.966E-01 4.418E-01 3.966E-01 4.418E-01 1 IL-1 beta

193 Immune response_IL-17 signaling pathways 60 3.966E-01 4.418E-01 3.966E-01 4.418E-01 1 IL-1 beta

194 Transcription_Role of VDR in regulation of genes involved in osteoporosis 61 4.017E-01 4.452E-01 4.017E-01 4.452E-01 1 IL-1 beta

195 Transcription_Sirtuin6 regulation and functions 64 4.167E-01 4.524E-01 4.167E-01 4.524E-01 1 LDLR

196 Immune response_IL-15 signaling 64 4.167E-01 4.524E-01 4.167E-01 4.524E-01 1 UFO

197 Glutathione metabolism 64 4.167E-01 4.524E-01 4.167E-01 4.524E-01 1 MGST

198 Development_Regulation of epithelial-to-mesenchymal transition (EMT) 64 4.167E-01 4.524E-01 4.167E-01 4.524E-01 1 IL-1 beta

199 Development_Role of IL-8 in angiogenesis 65 4.216E-01 4.532E-01 4.216E-01 4.532E-01 1 LDLR

200 Glutathione metabolism / Human version 65 4.216E-01 4.532E-01 4.216E-01 4.532E-01 1 MGST

201 Role of Tissue factor-induced Thrombin signaling in cancerogenesis 66 4.264E-01 4.561E-01 4.264E-01 4.561E-01 1 Tissue factor

202 Mucin expression in CF airways 69 4.408E-01 4.692E-01 4.408E-01 4.692E-01 1 IL-1 beta

203 Transcription_Effect of Folic acid on genome stability 70 4.455E-01 4.695E-01 4.455E-01 4.695E-01 1 TYSY

204 Glutathione metabolism / Rodent version 70 4.455E-01 4.695E-01 4.455E-01 4.695E-01 1 MGST

205 Transport_Clathrin-coated vesicle cycle 71 4.502E-01 4.721E-01 4.502E-01 4.721E-01 1 Syntaxin 16

206 Immune response_T regulatory cell-mediated modulation of antigen-presenting cell functions 72 4.548E-01 4.724E-01 4.548E-01 4.724E-01 1 MHC class II

207 Reproduction_GnRH signaling 72 4.548E-01 4.724E-01 4.548E-01 4.724E-01 1 Secretogranin II

208 Cell adhesion_Integrin inside-out signaling in T cells 74 4.640E-01 4.796E-01 4.640E-01 4.796E-01 1 MHC class II

209 Chemotaxis_Leukocyte chemotaxis 75 4.685E-01 4.819E-01 4.685E-01 4.819E-01 1 MHC class II

210 Blood coagulation_GPIb-IX-V-dependent platelet activation 76 4.730E-01 4.842E-01 4.730E-01 4.842E-01 1 Alpha-actinin

211 Aminoacyl-tRNA biosynthesis in mitochondrion 81 4.948E-01 5.042E-01 4.948E-01 5.042E-01 1 IARS2

212 Transport_Intracellular cholesterol transport 85 5.116E-01 5.189E-01 5.116E-01 5.189E-01 1 LDLR

213 Sphingolipid metabolism 91 5.358E-01 5.409E-01 5.358E-01 5.409E-01 1 DES1

214 Sphingolipid metabolism / Human version 92 5.398E-01 5.423E-01 5.398E-01 5.423E-01 1 DES1

215 Oxidative phosphorylation 105 5.878E-01 5.878E-01 5.878E-01 5.878E-01 1 COX Vb

**Supplementary file 1D:** Primer sequences for RT-qPCR analysis; clone ID and catalog numbers for shRNAs (Open Biosystems); antibodies used; source and concentration of chemical inhibitors used

| **Application** | **Gene symbol** | **Forward primer (5′-3′)** | **Reverse primer (5′-3′)** |
| --- | --- | --- | --- |
| RT-qPCR | *AURKB* | AGCCACGATCATGGAGGAGT | GTAGTCCAGGGTGCCACACA |
|  | *CCNE1* | GGAAGAGGAAGGCAAACGTG | TTGTCAGGTGTGGGGATCAG |
|  | *CDC45* | TCCGATTTCCGCAAAGAGTT | TGGAACAAGGCCTGAAGGAT |
|  | *CDK1* | CCGCCCTTTCCTCTTTCTTT | CAATCGGGTAGCCCGTAGAC |
|  | *CENPA* | ACCTTACATGCAGGCCGAGT | GCAAAGTCCAGACAGCATCG |
|  | *CKS1B* | TTGGGAGTTGCTTGGAGGTT | GGGACCAGCTTGGCTATGTC |
|  | *DSN1* | TCGGAAAGAGAAGCATGCAA | TCAAAGGCAACGAGCAGAAA |
|  | DUSP6 | TTGAGACGCTCGCTGTTTGT | AGCCGCTGGCTCTTAGTGTC |
|  | *E2F1* | TCCCTCCTGCAGTGTCTGAA | GGCCCAAACGTCATCAAAAT |
|  | *E2F2* | GGCCCATCCAGTCTTCTGAC | CCCTGGAACATGGACAGTGA |
|  | *E2F3* | GCCTCTGCAAGGAGCTTTGT | AGAGAGCCCTTTCCCCTGTC |
|  | *E2F4* | TGTGAGTGGTCCCATTGAGG | GGGCAGAGGTGGAGGTGTAG |
|  | *E2F5* | TAAGCCCGTGGTTTTTCCTG | CTGCAGAGCCTGGCTTCTTT |
|  | *E2F6* | GGGGATGAAGTGTGCTCCTC | CAGTACGACCCACCAGTCCA |
|  | *E2F7* | CTGTTGCTCAGACGGACCTG | GCTGAGGGTGCAGATGACAG |
|  | *E2F8* | AACCCAAGCTCAGCCATTGT | TTGGGTGTCACAGGAACAGG |
|  | *ETV5* | TCTTGATGACCCAGCCAATG | GAAGAGGGCATCTGGGTCAC |
|  | *FANCD2* | AAATTGCTTCCCTTGCCAGA | TTCTGGGACACCAACACCAG |
|  | *FBXO5* | TCAGAAATCAGCAGCCCAGA | TGAGGAGCTTGCCATCTGAA |
|  | *FOSL1* | GCCACCCTAGCCAATGTCTC | AAACAGTGGGCAGCTTTGGT |
|  | *IFI27* | ACGGTGAGGTCAGCTTCACA | AACTACGGCAGAGCCAGAGG |
|  | *IFI6* | CTGGTCTGCGATCCTGAATG | AGAGGTTCTGGGAGCTGCTG |
|  | *IKKβ* | AGGTCCGTGGTCCTGTCAGT | TAGGGCCGTGAAACTCTGGT |
|  | *KPNA2* | ATACACCAGCTGCCCGTCTT | TACAGTGCCCTGGTTGTTGC |
|  | *LDLR* | ATGGAAGAACTGGCGGCTTA | GGGACTCCAGGCAGATGTTC |
|  | *MCM10* | CCTTGGACAGACTCCCGAAC | AGCCAATCCTTTGCTTTCCA |
|  | *MCM3* | TGTGGCCAAAATCATCAAGC | GGACCAACTCCACAGCTTCC |
|  | *MX1* | TTAAGCCTTGGACCGCAGTT | GCTGTTCTCCTGCACCTCCT |
|  | *NCAPG2* | AGGACGGAAGGGAGAAGGAG | TCTGGAAGCACAGAGGCAAA |
|  | *NF1* | TGTGGAGCACACCCAGCAAT | TTTCGGGCCCCTGTTTTCTT |
|  | *NF-κB* | ATTCTGGTGGGGTGTGTTCC | GGGACCCCAGAGTTCCCTAC |
|  | *NRAS* | GCCACTTTCAAGCTGCACTG | GAGCTGGGGAAGTAGCAGGA |
|  | *ORC6* | ACGCCTGTAATCCCAGCACT | CAATCTCGGCTCACTGCAAC |
|  | *PKMYT1* | GTGACTTCGGACTGCTGGTG | ATGTTGCATGCCACTTCCAG |
|  | *SMC4* | CGACAATAAGATGGCGGTGA | ACTCGAAAATGGCGCCTAAA |
|  | *SPC25* | ACAGTCCCCGTTGTGTGATG | CCAACCCAAATGTCCAACAA |
|  | *SPRY2* | AGTCGTCTCCAGCTCCGAAC | CTCTGAGCTCTGGCCTCCAT |
|  | *STAT1* | CGGGTAGTTTCGCTTTCCTG | GCTACGCACAGCACGTTAGG |
|  | *TYMS* | GACAGCCTGGGATTCTCCAC | TGGCAGGACAGCTCACTGTT |
|  | *ZWILCH* | AAACTGCGATCGCTTTGGAT | AAAGGACCTCTGGGCTCTCC |
| CHIP assay |  |  |  |
|  | IFI6 Promoter *NF-κB* ChIP | TACTAATGATTGACTGATGGCC | CCACTGGTGATCAACTTGACTT |
|  | MCM10 Promoter E2F2 ChIP | CGGGATTCGCTGGCGTAGC | GAAGGAGGCTCAGATGCCCA |
|  | | | |
|  | **Gene symbol** | **Clone ID** | **Catalog number** |
| shRNAs | *IFI6* | V3LHS_402972 | RHS4430-101099309 |
|  |  | V3LHS_402973 | RHS4430-101099309 |
|  | *E2F2* | TRCN0000013798 | RHS3979-9581487 |
|  |  | TRCN0000013799 | RHS3979-9581488 |
|  | *RELA*  *(NF-κB-p65)* | TRCN0000014687 | RHS3979-9582376 |
|  |  | TRCN0000014683 | RHS3979-9582372 |
|  | *STAT1* | TRCN0000004264 | RHS3979-9572997 |
|  |  | TRCN0000004266 | RHS3979-9572999 |
|  | NF1 | TRCN0000039714 | RHS3979-9607103 |
|  |  | TRCN0000039715 | RHS3979-9607104 |
|  | IKKβ | V3LHS_390159 | RHS4430-101169321 |
|  |  | V3LHS_390160 | RHS4430-101169496 |
|  | | | |
|  | **Protein symbol** | **Antibody source** | **Dilution** |
| Immunoblot/ | *IFI6* | Sigma-Aldrich | 1:1000 |
| ChIP | *E2F2 (for western blot)* | Santa Cruz Biotechnology, Inc. | 1:250 |
|  | *E2F2 (for ChIP)* | Santa Cruz Biotechnology, Inc. | 2 μg |
|  | H3K9-Ac | Millipore | 1:500 |
|  | *NF-κB (for ChIP)* | Cell Signaling | 10 μl |
|  | *NF-κB (RELA/p65)* | Cell Signaling | 1:100 |
|  | STAT1 | Invitrogen | 1;200 |
|  | STAT1 (for ChiP) | Invitrogen | 5 μl |
|  | Phospho-ERK | Cell signaling | 1:100 |
|  | Total-ERK | Cell signaling | 1:100 |
|  | NF1 | Santa Cruz Biotechnology, Inc. | 1:250 |
|  | IkBα | Cell signaling | 1:100 |
|  | p-IKBα (Ser32) | Cell signaling | 1:100 |
|  | IKKβ | Cell signaling | 1:100 |
|  | Phosphor-H2A.X | Cell signaling | 1:100 |
|  | H2A.X | Cell signaling | 1:100 |
|  | Cleaved Caspase 3 | Cell signaling | 1:100 |
|  | IdU | BD BioScience | 1:25 |
|  | CldU | Abcam | 1:400 |
|  | Actin | Cell signaling | 1:200 |
|  | | | |
|  | **Compounds** | **Concentration** | **Source** |
|  | Aphidicolin | Different concentrations as indicated | Sigma-Aldrich |
|  | Camptothecin | Different concentrations as indicated | Sigma-Aldrich |
|  | HydroxyUrea | Different concentrations as indicated | Sigma-Aldrich |
|  | Trametinib | Different concentrations as indicated | Selleck Chemicals |
|  | IdU | 25 µM | Sigma-Aldrich |
|  | CldU | 250 µM | Sigma-Aldrich |
