# Supplementary material for: Interferon alpha-inducible protein 6 regulates NRASQ61K-induced melanomagenesis and growth
Source: eLife. 2016 Sep 8;5:e16432. doi: 10.7554/eLife.16432 (PMC5031487; doi:10.7554/eLife.16432)
Supplement: Supplementary file 2. — DOI: http://dx.doi.org/10.7554/eLife.16432.032 [file elife-16432-supp2.docx]

**Supplementary File Legend**

**Supplementary File 2.** Analysis of MAP kinase regulated and BRAF-signature genes for correlation with BRAF/NRAS/NF1 alteration status using melanoma TCGA dataset. (mutations, copy number alterations and mRNA expression at the z-score threshold of ±2.0 for indicated gene were analyzed for 287 available melanoma sample data in relation to with BRAF/NRAS/NF1 alteration status via cbioportal: http://www.cbioportal.org/).

**Supplementary File 2.** Analysis of MAP kinase regulated and BRAF-signature genes for correlation with BRAF/NRAS/NF1 alteration status using melanoma TCGA dataset. (mutations, copy number alterations and mRNA expression at the z-score threshold of ±2.0 for indicated gene were analyzed for 287 available melanoma sample data in relation to with BRAF/NRAS/NF1 alteration status via cbioportal: http://www.cbioportal.org/).

| **Joseph et al., 2010, PNAS**  **PMID:**  **2068238** | **Gene ID** | **Downregulated after RAFi and MEKi treatment** | **Upregulated**  **after RAFi and MEKi treatment** | **Significant Tendency towards co-occurrence/BRAF alterations** | **Significant Tendency towards mutual exclusivity/**  **BRAF alterations** | **p-value** | **Significant Tendency towards co-occurrence/**  **NRAS alterations** | **Significant Tendency towards mutual exclusivity/**  **NRAS alterations** | **p-value** | **Significant Tendency towards co-occurance/NF1-deficiency** | **Significant Tendency towards mutual exclusivity**  **/NF1-deficiency** | **p-value** |
| --- | --- | --- | --- | --- | --- | --- | --- | --- | --- | --- | --- | --- |
|  | DUSP6 | Yes | No | Yes | No | 0.02 | No | No |  | No | No |  |
|  | SPRY2 | Yes | No | No | No |  | No | No |  | No | No |  |
|  | IER3 | Yes | No | No | No |  | Yes |  | 0.021 | No | No |  |
|  | SPRY4 | Yes | No | No | No |  | No | No |  | No | No |  |
|  | FOSL1 | Yes | No | No | No |  | No | No |  | No | No |  |
|  | RND3 | Yes | No | No | No |  | No | No |  |  |  |  |
|  | EGR1 | Yes | No | No | Yes | <.0.001 | Yes | No | <0.001 | No | No |  |
|  | HES1 | Yes | No | No | No |  | No | No |  | No | No |  |
|  | GDF15 | Yes | No | No | No |  | No | No |  | No | No |  |
|  | CCND1 | Yes | No | No | No |  | No | No |  | No | No |  |
|  | SH2B3 | Yes | No | Yes | No | 0.027 | No | No |  | No | No |  |
|  | ETV5 | Yes | No | No | No |  | No | No |  | No | No |  |
|  | MYC | Yes | No | No | No |  | No | No |  | No | No |  |
|  | MANSC1 | Yes | No | No | No |  | No | No |  | No | No |  |
|  | ST8SIA1 | Yes | No | Yes | No | 0.011 | No | Yes | 0.049 | No | No |  |
|  | MAFF | Yes | No | No | Yes | 0.005 | No | No |  | No | No |  |
|  | SLC16A6 | Yes | No | No | No |  | No | No |  | No | No |  |
|  | PHLDA2 | Yes | No | Yes | No | 0.032 | No | No |  | No | No |  |
|  | ETV1 | Yes | No | No | No |  | No | No |  | No | No |  |
|  | DUSP4 | Yes | No | No | No |  | No | No |  | No | No |  |
|  | SLC20A1 | Yes | No | Yes | No | 0.007 | No | Yes | 0.011 | No | No |  |
|  | PPAP2B (PLPP3) | Yes | No | No | No |  | No | No |  | Yes | No | 0.047 |
|  | INPP5F | Yes | No | No | Yes | 0.028 | No | No |  | No | No |  |
|  | ELK3 | Yes | No | No | No |  | No | No |  | No | No |  |
|  | IER2 | Yes | No | No | Yes | 0.016 | Yes | No | 0.011 | No | No |  |
|  | SLC43A3 | Yes | No | No | No |  | No | No |  | No | No |  |
|  | HMGA2 | Yes | No | No | No |  | No | No |  | No | No |  |
|  | B4GALT6 | Yes | No | No | No |  | No | No |  | No | No |  |
|  | STK17A | Yes | No | Yes | No | 0.016 | No | Yes | 0.022 | No | No |  |
|  | TFAP2C | Yes | No | Yes | No | 0.017 |  | No | No | No | No |  |
|  | SPRED2 | Yes | No | No | No |  | No | No |  | No | No |  |
|  | SPRY1 | Yes | No | No | No |  | No | No |  | Yes | No | 0.006 |
|  | PMAIP1 | Yes | No | No | No |  | No | No |  | No | No |  |
|  | VDR | Yes | No | No | No |  | No | No |  | No | No |  |
|  | SEMA4C | Yes | No | No | No |  | No | No |  | Yes | No | 0.019 |
|  | TNFAIP3 | Yes | No | No | No |  |  |  |  |  |  |  |
|  | HAS2 | No | Yes | No | Yes | 0.049 | No | No |  | No | No |  |
|  | ID2IIID2B | No | Yes |  |  |  |  |  |  |  |  |  |
|  | ID2 | No | Yes | No | No |  | No | No |  | No | No |  |
|  | SEMA6A | No | Yes | No | No |  | No | No |  | No | No |  |
|  | PPARGC1A | No | Yes | No | No |  | No | No |  | No | No |  |
|  | SEMA3C | No | Yes | No | No |  | No | No |  | No | No |  |
|  | STON1 | No | Yes | No | No |  | No | No |  | No | No |  |
|  |  |  |  |  |  |  |  |  |  |  |  |  |
| **Kannengiesser et al., 2008;**  **PMID:**  **19383316** | **Gene ID** | **All genes**  **upregulated in BRAF-mutant melanoma** |  | **Significant Tendency towards co-occurrence/BRAF alterations** | **Significant Tendency towards mutual exclusivity/**  **BRAF alterations** | **p-value** | **Significant Tendency towards co-occurrence/**  **NRAS alterations** | **Significant Tendency towards mutual exclusivity/**  **NRAS alterations** | **p-value** | **Significant Tendency towards co-occurance/NF1-deficiency** | **Significant Tendency towards mutual exclusivity**  **/NF1-deficiency** | **p-value** |
|  | SYPL (SYPL1) |  |  | Yes | No | <0.001 | No | Yes | 0.011 | No | No |  |
|  | CD63 |  |  | No | No |  | No | No |  | No | No |  |
|  | ETV1 |  |  | No | No |  | No | No |  | No | No |  |
|  | MGC2941 |  |  |  |  |  |  |  |  |  |  |  |
|  | HRMT1L1 (PRMT2) |  |  | No | No |  | No | No |  | No | No |  |
|  | GUSB |  |  | No | No |  | No | No |  | No | No |  |
|  | DPH2L1 (DPH1) |  |  | No | No |  | No | No |  | No | No |  |
|  | S100A13 |  |  | No | No |  | No | No |  | No | No |  |
|  | CSPG4 |  |  | No | No |  | No | No |  | No | No |  |
|  | ARPC1B |  |  | No | No |  | No | No |  | No | No |  |
|  | TMP21-II (TMED10P1) |  |  | No | No |  | No | No |  | No | No |  |
|  | DAD1 |  |  | No | No |  | No | No |  | Yes | No | <0.001 |
|  | LRPAP1 |  |  | No | No |  | No | Yes | 0.042 | No | No |  |
|  | MAGED2 |  |  | No | No |  | No | No |  | No | No |  |
|  | BC038098 |  |  |  |  |  |  |  |  |  |  |  |
|  | PTG2 |  |  |  |  |  |  |  |  |  |  |  |
|  | BC037430 |  |  |  |  |  |  |  |  |  |  |  |
|  | BC028093 |  |  |  |  |  |  |  |  |  |  |  |
|  | PMS2L4 (PMS2P4) |  |  | Yes | No | 0.002 | No | Yes | 0.001 | No | No |  |
|  | NDUFB2 |  |  | Yes | No | <0.001 | No | Yes | <0.001 | No | No |  |
|  | MIA |  |  | Yes | No | 0.005 | No | Yes | 0.006 | No | No |  |
|  | MRPS6 |  |  | Yes | No | 0.007 | No | No |  | No | No |  |
|  | SERPINE2 |  |  | No | No |  | No | No |  | No | No |  |
|  | LAP1B (TOR1AIP1) |  |  | No | No |  | Yes | No | 0.032 | Yes | No | 0.035 |
|  | HSPA6 |  |  | No | No |  | No | No |  | No | No |  |
|  | FLJ20452 |  |  |  |  |  |  |  |  |  |  |  |
|  | FLJ38993 |  |  |  |  |  |  |  |  |  |  |  |
|  | HSPC015 (DCPS) |  |  | No | No |  | No | Yes | 0.012 | No | No |  |
|  | HUS1 |  |  | Yes | No | 0.002 | No | Yes | 0.004 | No | No |  |

** For the genes highlighted in gray in the Gene ID column, no information could be retrieved from the cBIO portal analysis because the gene IDs were not recognized as valid gene IDs.
